# Prevalence of Behavioral and Psychological Symptoms in Patients With Cognitive Decline Before and During the COVID-19 Pandemic

**DOI:** 10.3389/fpsyt.2022.839683

**Published:** 2022-03-07

**Authors:** Yujiro Kuroda, Taiki Sugimoto, Nanae Matsumoto, Kazuaki Uchida, Yoshinobu Kishino, Claudia Kimie Suemoto, Takashi Sakurai

**Affiliations:** ^1^Department of Prevention and Care Science, Center for Development of Advanced Medicine for Dementia, National Center for Geriatrics and Gerontology, Obu, Japan; ^2^Center for Comprehensive Care and Research on Memory Disorders, National Center for Geriatrics and Gerontology, Obu, Japan; ^3^Department of Public Health, Graduate School of Health Sciences, Kobe University, Kobe, Japan; ^4^Department of Cognitive and Behavioral Science, Nagoya University Graduate School of Medicine, Nagoya, Japan; ^5^Division of Geriatrics, University of São Paulo, São Paulo, Brazil

**Keywords:** behavioral and psychological symptoms (BPSD), social distancing (vaccine), dementia–Alzheimer's disease, coronavirus disease (COVID-19), mild cognitive impairment (MCI)

## Abstract

**Objective:**

Preventive measures to limit the spread of COVID-19 are essential, but often cause social isolation, affecting the physical and mental health of older adults. Patients with dementia are likely to have worsening behavioral and psychological symptoms of dementia (BPSD) owing to pandemic restrictions. To examine this, we described BPSD before and during the COVID-19 pandemic.

**Methods:**

We identified patients at a memory clinic in Japan between October 2018 and December 2019 (15 months before the pandemic began, *n* = 1,384) and between April 2020 and June 2021 (15 months after the State of Emergency was declared; *n* = 675 patients). A propensity score was used to match 576 patients from each group. The Mini-Mental State Exam was used to classify cognitive function into mild and moderate/severe. Dementia Behavioral Disturbance Scale was used to evaluate BPSD. The association between BPSD before and during the pandemic was evaluated using binomial logistic regression models.

**Results:**

The levels of frequent night waking were higher in individuals before the pandemic than in those evaluated during the pandemic in both the mild group [adjusted odds ratio (AOR) = 1.82, 95% CI 1.02–3.23] and the moderate/severe group (AOR = 1.96, 95% CI 1.19–3.23). During the pandemic, physical attacks were higher in the mild group (AOR = 4.25, 95% CI 1.12–16.07), while night wandering was higher in the moderate/severe group (AOR = 2.22, 95% CI 1.03–4.81).

**Conclusion:**

In patients with cognitive impairment, some BPSD were more prevalent during the pandemic, depending on dementia severity. The findings pertaining to the higher frequency of sleep disturbance and aggressiveness during COVID-19 should be used to guide BPSD screening in patients with dementia and to provide evidence-based interventions.

## Introduction

Outbreaks of COVID-19 caused by severe acute respiratory syndrome coronavirus 2 (SARS-CoV-2) continue to be the most important issue in health risk management in Japan since the first case was found there in January 2020. The International Epidemiology Association's Dictionary of Epidemiology defines a pandemic as “an epidemic occurring worldwide, or over a very wide area, crossing international boundaries and usually affecting a large number of people” (1). The complexity of this pandemic is that while minimizing the infection risk is extremely important, the most vulnerable members of society, such as patients with dementia and those requiring long-term care, are likely to be affected by long-term measures, such as social distancing.

Systematic reviews have shown that confinement and isolation are effective for infection control, including in the COVID-19 pandemic (2). However, in past outbreaks such as SARS and MERS, a public health challenge also emerged in that the countermeasure—long-term “quarantine”—compromised mental health and increased psychological symptoms, especially those related to stress responses such as anxiety, depression, and distress (3). In addition, increased vigilance due to fear of contagion and grief over the loss of family and friends due to the disaster can undermine mental health and wellbeing (4). These findings are based on the general population, but there are very few findings on the socially vulnerable elderly and people with dementia (5, 6). People with dementia are frail, often dependent on caregivers for activities of daily living (7), and they are frequently in need of nursing care and social services (e.g., dementia café, daycare, and residential care) (8, 9). Prolonged isolation keeps them away from these protective factors and negatively affects their behavior and psychology (i.e., behavioral and psychological symptoms of dementia, BPSD), thus amplifying the burden on caregivers (10). In a telephone survey of patients with dementia and mild cognitive impairment in Italy, 32% showed deterioration in memory and orientation, and 8% showed a functional decline in daily life, described mainly in terms of reduced levels of independence in personal care and housekeeping (11). BPSD, mainly agitation/aggression, apathy, and depression, worsened or developed in more than 50% of patients, mainly in dementia patients (11). In a survey of dementia patients and their families after COVID-19 in Japan, 39% of medical and nursing facilities and 38% of care support specialists reported that their dementia patients were affected in some way, such as the appearance or worsening of BPSD, decline in cognitive function, and decline in physical activity (12). In a survey of care support specialists and physicians certified in dementia care by academic societies in Japan, 40% of dementia patients experienced a worsening of their condition, most frequently a worsening of cognitive function (47%) and an increase or worsening of BPSD (46%).

Previous studies have suggested that BPSD could worsen or develop in patients with dementia after the COVID-19 pandemic (11–13), but their designs did not include the normal condition (i.e., before COVID-19). In addition, the severity of dementia is closely related to the development of BPSD (14, 15). Therefore, describing BPSD before and after the COVID-19 pandemic as well as considering dementia severity could provide a more detailed context and clinical insights. The purposes of this study were to describe the prevalence of behavioral and psychological symptoms, mainly BPSD, before and during the COVID-19 pandemic in patients with cognitive decline and to obtain knowledge to improve the quality of medical care for patients with dementia during and after COVID-19.

## Methods

### Study Cohort

Participants were patients of the memory clinic of the National Center for Geriatrics and Gerontology (NCGG) in Aichi, Japan, from October 2018 to December 2019 (15 months before the onset of the COVID-19 pandemic; “before COVID-19”, *n* = 1,382) or from April 2020 to June 2021 (15 months after the declaration of the COVID-19 emergency; “during COVID-19”, *n* = 675 patients), and had a dementia-related diagnosis according to the criteria of the National Institute on Aging-Alzheimer's Association workgroups (16, 17). Specifically, mild cognitive impairment (MCI) due to Alzheimer's disease (AD) (16) and dementia were classified as either probable or possible AD (17), probable or possible dementia with Lewy bodies and Parkinson's disease (DLB/PD) (18, 19), and vascular dementia (VaD) (20).

The period from the beginning of January 2020 to the end of March 2020 was excluded because it was the early stage of the pandemic, and the relationship between the disease and people's behavior was unclear. Only the first assessment of those with multiple assessments was included (before COVID-19 = 982, during COVID-19 = 615, with no overlap between the groups). We selected 883 before-COVID-19 and 576 during-COVID-19 participants who had also completed the Dementia Behavioral Disturbance Scale (DBD) (21), the primary outcome of this study. To ensure that the analysis took into account the characteristics of the participants before and during COVID-19, the 2 groups were matched using propensity scores. To calculate the propensity score, age, gender, and type of dementia (13) were used as the predictor variables to estimate the probability of belonging to the before- and during-COVID-19 groups. We matched participants with similar propensity scores at 1:1 to create a dataset of 576 individuals in each group (Supplementary Figure). The study was conducted after the participants approved that their data would be included in the study and the Ethics Committee of the NCGG approved the study protocol.

### Variables

#### Behavioral and Psychological Symptoms of Dementia

BPSD was assessed with the DBD developed by Baumgarten et al. (21). The DBD consists of 28 observable behaviors related to dementia, and the frequency of each item is rated by a primary caregiver on a scale of 0 to 4 (0 = never, 1 = rarely, 2 = sometimes, 3 = frequently, 4 = always), with higher scores indicating greater severity of BPSD. DBD includes various domains of observable behavioral disorders such as passivity, agitation, eating disturbances, aggressiveness, diurnal rhythm disturbances, and sexual disinhibition. The reliability and validity of the Japanese version of the DBD have been established (22), and the scale is widely used internationally in the field of dementia. The DBD was completed by the primary caregiver (patient's spouse or child, 90.5%) independent of the patient and collected by an outpatient health care provider. Several measures have been developed to assess BPSD [e.g., Neuropsychiatric Inventory (NPI) (23) and DBD], and there are differences in the way BPSD is assessed by medical professionals and caregivers. These measures have been widely used mainly for dementia patients, but research that assesses behavioral and psychological symptoms in people with MCI has also been published (24, 25).

#### Sociodemographic and Clinical Variables

Cognitive function in the elderly was assessed by use of the Mini-Mental State Exam (MMSE) (26). Scores range from 0 to 30, with higher scores reflecting a higher level of global cognitive performance. In this study, total MMSE scores were classified into three groups: mild (MMSE 21–30 points, equivalent to Clinical Dementia Raring; CDR 0–1), moderate (11–20 points, equivalent to CDR 2), and severe (0–10, equivalent to CDR 3), according to studies that examined the relationship between CDR and MMSE (27). The results showed unbalanced groups, with 55.6% placed in the mild, 46.2% in the moderate, and only 4.1% in the severe categories. To make the groups more balanced, the moderate and severe groups were combined into one category.

Depressive symptoms were assessed with the 15-item Geriatric Depression Scale (GDS), with higher scores indicating greater depressive symptoms (28). In addition, the ability to walk and balance was assessed with the Timed Up and Go Test (TUG) (29). Other information such as gender, age, education, marital status, living environment, comorbidity (diabetes mellitus, hypertension, dyslipidemia, cardiac disease, and stroke), polypharmacy (5 or more prescribed medications) (30), and body mass index were obtained from medical records.

#### Informant-Based Variables

Using a questionnaire, the primary caregiver reported the following about the patient: (a) Basic activities of daily living (ADL), assessed by the Barthel Index (31), and Instrumental ADL (IADL), assessed by the Lawton Index (32), with higher scores indicating better status; (b) Financial status was determined by the need for assistance on a 4-point scale (25); (c) Use of care services: home care service, daycare, or residential care; (d) Status of care needs: 7 levels of Long-term Care Insurance System (LTCI) certification, “requiring support” levels 1 and 2, and “requiring long-term care” levels 1 to 5 (33); (e) Patient lifestyle: light exercise/physical training, regular exercise/sport, regular drinking, regular smoking, quality of sleep, napping, weight loss, and fatigue. The LTCI certification was classified into “mild” (“requiring support” levels 1 and 2 and “requiring long-term care” level 1) or “severe” (“requiring long-term care” levels 2 to 5) based on studies by Saito et al. (33) and Fujiwara et al. (34).

#### Statistical Analysis

To analyze socioeconomic status and lifestyle-related variables, physical functioning, and psychological functioning between the mild and moderate/severe groups before and during the COVID-19 pandemic, we used independent-sample *t*-tests for continuous variables and chi-squared tests for categorical variables. Next, we analyzed the prevalence of each DBD item in the 2 groups before and during COVID-19. The presence of BPSD was defined by responses of “sometimes,” “often,” or “always,” (21) and the prevalence before and during the pandemic were analyzed using the chi-squared test stratified by dementia severity. Items that were significant in the univariate analysis were selected for further multivariate analysis to explore the association between DBD and the period related to the COVID-19 pandemic. Binomial logistic regression analysis was performed with each item of the DBD as an outcome and before and during COVID-19 as the explanatory variable. Odds ratios (ORs) and confidence intervals (Cis) were estimated. The models were adjusted for three sets of variables: (a) socioeconomic status (financial burden, living alone, and educational history); (b) physical functioning and use of medical and nursing care services (IADL, polypharmacy, and LTCI certification); and (c) cognitive and psychological functioning (MMSE and GDS).

**Figure 1:**
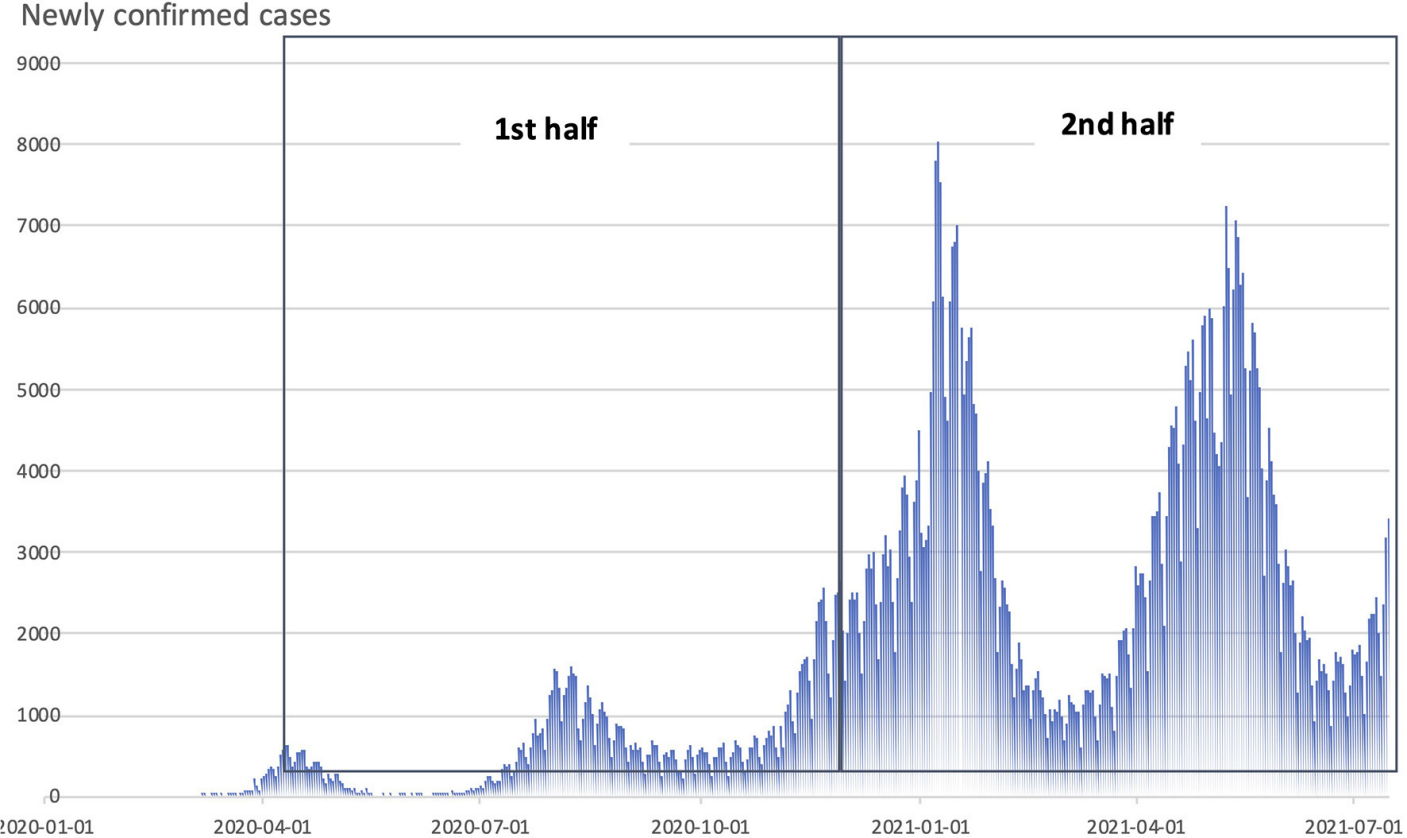
Monthly trends in the number of new COVlD-19 infections in Japan and the period of the pandemic covered by this study (divided into first half and second halves: the first half was set at 7½ months, from April 2020 to mid-October 2020,and the second from then until the end of June 2021).

Because the during-COVID-19 observation period was 15 months, during which time the state of emergency was declared and various infection prevention measures were taken, we conducted a subanalysis of BPSD items in the early (first half) and late (second half) of the pandemic period (Figure 1). All analyses were carried out in SPSS v. 27.0 (IBM Corporation, Armonk, NY, USA). *P*-values < 0.05 were considered statistically significant.

## Results

The analysis of MMSE scores before and during the pandemic showed no difference between the two groups (20.96 ± 5.15 and 20.27 ± 5.20, respectively; *p* = 0.912). The Mild group included 327 before-COVID-19 patients and 313 during-COVID-19 patients. The moderate/severe group included 249 and 261, respectively. Age group, gender, clinical diagnosis, and comorbidities did not differ between the “before” and “during” groups (Table 1). But the following variables did differ between them: In the mild group, significantly more people were married (*P* = 0.003), had more than 12 years of education (*P* = 0.047), and were less likely to live alone (*P* = 0.032) during COVID-19 than before. In the moderate/severe group, more people had worse sleep (*P* = 0.040), with higher scores in the GDS (*P* < 0.001). Further, we observed a trend of less residential care use during the COVID-19 pandemic (*P* = 0.050).

**Table 1:** Relationship between basic characteristics and lifestyle before and during COVID-19 according to the severity of cognitive impairment.

	**Total group**			**Mild group** **(MMSE 21–30)**			**Moderate/severe group** **(MMSE 11–20)**		
			***P*-value**			***P*-value**			***P*-value**
	**Before**	**During**		**Before**	**During**		**Before**	**During**	
	**(*n* = 576)**	**(*n* = 576)**		**(*n* = 327)**	**(*n* = 313)**		**(*n* = 249)**	**(*n* = 261)**	
	***n* (%)**	***n* (%)**		***n* (%)**	***n* (%)**		***n* (%)**	***n* (%)**	
Socioeconomic status									
Age group									
Under 64	28 (4.9)	30 (5.2)	0.884	21 (6.4)	17 (5.4)	0.865	7 (2.8)	13 (5.0)	0.321
65–74	99 (17.2)	104 (18.1)		63 (19.3)	60 (19.2)		36 (14.5)	44 (16.8)	
75 and over	449 (78.0)	442 (76.7)		243 (74.3)	236 (75.4)		206 (82.7)	205 (78.2)	
Gender (female)	359 (62.3)	338 (58.5)	0.206	188 (57.5)	174 (55.6)	0.633	171 (68.7)	162 (61.8)	0.115
Marital status (married)	557 (96.7)	563 (98.1)	0.195	315 (96.3)	310 (99.7)	**0.003**	242 (97.2)	252 (96.2)	0.625
Education (12 y or above)	258 (44.9)	302 (52.5)	**0.011**	168 (51.4)	186 (59.4)	**0.047**	90 (36.4)	116 (44.4)	0.071
Living alone	97 (16.9)	75 (13.1)	0.082	56 (17.2)	35 (11.3)	**0.032**	41 (16.5)	40 (15.3)	0.718
Need for financial support	38 (6.6)	42 (7.4)	0.644	15 (4.6)	16 (5.2)	0.854	23 (9.3)	26 (10.1)	0.881
Use of care services									
Visiting home	19 (3.3)	10 (1.7)	0.131	9 (2.8)	5 (1.6)	0.420	10 (4.0)	5 (1.9)	0.194
Day care	119 (20.7)	106 (18.4)	0.373	48 (14.7)	41 (13.1)	0.570	71 (28.5)	64 (24.4)	0.316
Residential care	17 (3.0)	5 (0.9)	**0.016**	4 (1.2)	0 (0.0)	0.124	13 (5.2)	5 (1.9)	0.050
Needed support/Long-term care									
Certification (mild)	128 (22.3)	100 (17.6)	0.106	53 (16.4)	36 (11.6)	0.214	75 (30.1)	64 (25.0)	0.404
Certification (severe)	42 (7.3)	51 (9.0)		13 (4.0)	15 (4.8)	0.214	29 (11.6)	35 (13.7)	
Medical condition									
Clinical diagnosis									
MCI	232 (40.3)	237 (41.1)	0.771	204 (62.4)	206 (65.8)	0.335	28 (11.2)	31 (11.8)	0.443
AD	293 (50.9)	279 (48.4)		99 (30.3)	83 (26.5)		194 (77.9)	194 (74.0)	
DLB/PD	35 (6.1)	41 (7.1)		18 (5.5)	13 (4.2)		17 (6.8)	28 (10.7)	
VaD	16 (2.8)	19 (3.3)		6 (1.8)	11 (3.5)		10 (4.0)	8 (3.1)	
Polypharmacy (5 or above)	209 (36.3)	207 (35.9)	0.951	119 (36.4)	103 (32.9)	0.362	90 (36.1)	104 (39.7)	0.414
Comorbidity									
Diabetes mellitus	97 (53.0)	86 (47.0)	0.606	56 (17.7)	42 (13.4)	0.154	41 (17.1)	44 (16.9)	1.000
Hypertension	265 (49.7)	268 (50.3)	0.486	148 (46.7)	138 (44.1)	0.523	117 (48.8)	130 (49.8)	0.858
Dyslipidemia	120 (52.4)	109 (47.6)	0.209	75 (23.7)	62 (19.8)	0.248	45 (18.8)	47 (18.0)	0.908
Cardiac disease	93 (55.0)	76 (45.0)	0.134	60 (18.9)	42 (13.4)	0.066	33 (13.8)	34 (13.0)	0.896
Stroke	31 (43.7)	40 (56.3)	0.441	22 (6.9)	26 (8.3)	0.551	9 (3.8)	14 (5.4)	0.404
Lifestyle-related variables									
Light exercise/physical training	277 (48.6)	258 (45.0)	0.236	166 (51.2)	152 (48.7)	0.579	111 (45.1)	105 (40.4)	0.323
Regular exercise/sport	140 (24.5)	126 (22.0)	0.327	97 (29.8)	73 (23.4)	0.074	43 (17.6)	53 (20.3)	0.496
Regular drinking	92 (16.1)	98 (17.1)	0.692	57 (17.6)	59 (18.9)	0.682	35 (14.2)	38 (14.6)	1.000
Regular smoking	40 (7.0)	32 (5.6)	0.332	22 (6.8)	17 (5.4)	0.512	18 (7.3)	15 (5.7)	0.590
Quality of sleep (worse)	52 (9.2)	60 (10.5)	0.487	34 (10.6)	26 (8.4)	0.416	18 (7.4)	34 (13.1)	**0.040**
Napping (often)	57 (10.1)	80 (14.0)	**0.045**	29 (9.1)	34 (10.9)	0.507	28 (11.4)	45 (17.4)	0.058
Weight loss	88 (15.4)	99 (17.3)	0.380	51 (15.7)	53 (17.0)	0.669	37 (15.0)	46 (17.8)	0.404
Fatigue	94 (16.5)	90 (15.8)	0.748	58 (18.0)	40 (13.0)	0.099	36 (14.7)	50 (19.2)	0.194
				**Mean** **±SD**	**Mean** **±SD**		**Mean** **±SD**	**Mean** **±SD**	
Physical functioning									
IADL (Lawton Index Score)									
Male	3.6 ± 1.4	3.5 ± 1.4	0.587	4.0 ± 1.2	4.1 ± 1.1	0.595	2.8 ± 1.4	2.8 ± 2.5	0.698
Female	5.9 ± 2.1	5.9 ± 2.2	0.854	6.7 ± 1.6	6.8 ± 1.5	0.409	5.0 ± 2.3	5.0 ± 2.3	0.895
Barthel Index score	95.3 ± 10.8	94.0 ± 12.4	0.061	97.7 ± 5.9	97.0 ± 7.9	0.204	92.0 ± 14.4	90.5 ± 15.5	0.247
BMI	22.2 ± 3.5	22.1 ± 3.3	0.706	22.5 ± 3.5	22.4 ± 3.3	0.348	21.8 ± 3.6	21.8 ± 3.2	0.998
TUG	12.4 ± 4.6	12.2 ± 3.7	0.509	11.5 ± 3.6	11.4 ± 2.9	0.815	13.7 ± 5.5	13.4 ± 4.4	0.612
**Psychological functioning**									
GDS	3.1 ± 2.9	3.6 ± 3.1	**0.015**	3.1 ± 3.0	3.3 ± 2.9	0.251	3.0 ± 2.7	4.1 ± 3.1	**<0.001**

*P-values in bold indicate statistical significance*.

*AD, Alzheimer's disease; BMI, body mass index; DLB/PD, Lewy body disease/Parkinson's disease dementia; GDS, Geriatric Depression Scale; IADL, instrumental activities of daily living; MCI, mild cognitive impairment; MMSE, Mini-Mental State Examination; TUG, Timed Up and Go; VaD, vascular dementia*.

The five most prevalent BPSD items were as follows: “Asks the same question over and over again,” “Loses, misplaces, or hides things,” “Shows lack of interest in daily activities,” “Sleeps excessively during the day,” and “Hoards things for no obvious reason,” which were frequent in all the groups (Table 2). However, these items were more frequent in the moderate/severe group than in the mild group (specifically, for the question “Asks the same question over and over again,” 78.3% of the mild group vs. 94.0% of the moderate/severe group showed higher prevalence before COVID-19, and 78.3% of the mild group vs. 91.2% of the moderate/severe group showed higher prevalence during COVID-19). Whereas, items related to sexual disinhibition, specifically the items of “Makes inappropriate sexual advances” and “Exposes himself/herself indecently,” were not frequent, ranging from 0 to 1.6%. Urinary and fecal incontinence was also not frequent, ranging from 6.4 to 21.0% for urinary incontinence, and from 0 to 0.9% for fecal incontinence. Comparing prevalence before and during COVID-19 by severity, we found “before” and “after” differences in several items in patients with mild and moderate/severe dementia (Table 2), such as “Wakes up at night for no obvious reason” (6.9 vs. 12.2%) and “Physical attacks” (0.9 vs. 3.5%) in the mild group, and “Wakes up at night for no obvious reason” (15.9 vs. 28.9%), “Sleeps excessively during the day” (41.9 vs. 51.3%), “Paces up and down” (12.9 vs. 20.4%), “Wanders in the house at night” (5.7 vs. 11.2%), and “Gets lost outside” (6.5 vs. 11.9%) in the moderate/severe group (Table 2). Those items were further analyzed by multivariate analysis. In the unadjusted model, all of the items remained significant (Table 3); however, in the adjusted models, only “Wakes up at night for no obvious reason” (AOR = 1.82, 95% CI 1.02–3.23 in the mild group; AOR = 1.96, 95% CI 1.19–3.23 in the moderate/severe group), “Physical attacks” (AOR = 4.25, 95% CI 1.12–16.07 in the mild group), and “Wanders in the house at night” (AOR = 2.22, 95% CI 1.03–4.81 in the moderate/severe group) remained significant.

**Table 2:** Proportion of patients with behavioral disorders in each item of DBD: before and during COVID-19 pandemic, by severity of cognitive impairment.

	**Total group**			**Mild group** **(MMSE 21–30)**			**Moderate/severe group** **(MMSE 11–20)**		
	**Before**	**During**		**Before**	**During**		**Before**	**During**	
			***P*-value**			***P*-value**			***P*-value**
01. Asks the same question over and over again	490 (85.1)	484 (84.0)	0.684	256 (78.3)	245 (78.3)	1.000	234 (94.0)	239 (91.2)	0.244
02. Loses, misplaces, or hides things	433 (75.3)	416 (72.3)	0.283	221 (67.6)	199 (63.6)	0.318	212 (85.5)	216 (82.8)	0.467
03. Shows lack of interest in daily activities	285 (49.7)	288 (50.4)	0.813	136 (41.7)	125 (40.2)	0.747	149 (60.3)	161 (62.2)	0.715
04. Wakes up at night for no obvious reason	61 (10.8)	113 (19.9)	**<0.001**	22 (6.9)	38 (12.2)	**0.029**	39 (15.9)	74 (28.9)	**<0.001**
05. Makes unwarranted accusations	104 (18.1)	118 (20.5)	0.331	48 (14.7)	50 (16.0)	0.662	56 (22.6)	67 (25.7)	0.469
06. Sleeps excessively during the day	219 (38.2)	245 (42.6)	0.133	115 (35.3)	110 (35.1)	1.000	104 (41.9)	134 (51.3)	**0.041**
07. Paces up and down	44 (7.7)	70 (12.2)	**0.010**	12 (3.7)	17 (5.4)	0.343	32 (12.9)	53 (20.4)	**0.024**
08. Repeats the same action over and over again	68 (11.8)	81 (14.1)	0.255	23 (7.0)	26 (8.3)	0.556	45 (18.1)	54 (20.8)	0.502
09. Is verbally abusive, curses	91 (15.8)	104 (18.1)	0.346	43 (13.2)	49 (15.7)	0.430	48 (19.3)	54 (20.7)	0.740
10. Dresses inappropriately	101 (17.6)	102 (17.8)	0.938	40 (12.3)	29 (9.3)	0.252	61 (24.6)	72 (27.8)	0.421
11. Cries or laughs inappropriately	34 (5.9)	46 (8.0)	0.166	9 (2.8)	17 (5.4)	0.109	25 (10.0)	29 (11.2)	0.774
12. Refuses to be helped with personal care	110 (19.2)	124 (21.6)	0.341	55 (16.9)	51 (16.3)	0.915	55 (22.3)	73 (28.0)	0.153
13. Hoards things for no obvious reason	134 (23.3)	151 (26.4)	0.246	64 (19.6)	65 (20.9)	0.694	70 (28.3)	85 (32.6)	0.335
14. Moves arms or legs in a restless or agitated way	33 (5.7)	35 (6.1)	0.804	16 (4.9)	10 (3.2)	0.320	17 (6.9)	25 (9.6)	0.334
15. Empties drawers or closets	35 (6.1)	48 (8.4)	0.139	10 (3.1)	12 (3.9)	0.287	25 (10.0)	36 (13.8)	0.219
16. Wanders in the house at night	19 (3.3)	38 (6.7)	**0.014**	5 (1.5)	9 (2.3)	0.591	14 (5.7)	29 (11.2)	**0.037**
17. Gets lost outside	21 (3.7)	43 (7.5)	**0.005**	5 (1.5)	12 (3.9)	0.085	16 (6.5)	31 (11.9)	**0.046**
18. Refuses to eat	16 (2.8)	23 (4.0)	0.328	5 (1.5)	9 (3.1)	0.288	11 (4.4)	14 (5.4)	0.685
19. Overeats	92 (16.0)	82 (14.3)	0.459	46 (14.1)	34 (10.9)	0.234	46 (18.5)	48 (18.5)	1.000
20. Is incontinent of urine	73 (12.7)	77 (13.4)	0.793	21 (6.4)	26 (8.3)	0.368	52 (21.0)	50 (19.2)	0.658
21. Wanders aimlessly outside or in the house during the day	24 (4.2)	39 (6.8)	0.053	6 (1.8)	7 (2.2)	0.784	18 (7.2)	32 (12.3)	0.073
22. Physical attacks	10 (1.7)	22 (3.8)	**0.032**	3 (0.9)	11 (3.5)	**0.030**	7 (2.8)	11 (4.2)	0.475
23. Screams for no reason	11 (1.9)	17 (3.0)	0.258	4 (1.2)	9 (2.9)	0.167	7 (2.8)	8 (3.1)	1.000
24. Makes inappropriate sexual advances	6 (1.0)	2 (0.3)	0.287	3 (0.9)	0 (0.0)	0.249	3 (1.2)	2 (0.8)	0.679
25. Exposes himself/herself indecently	2 (0.3)	1 (0.2)	1.000	0 (0.0)	0 (0.0)	1.000	2 (0.8)	1 (0.4)	0.615
26. Destroys property or clothing	7 (1.2)	5 (0.9)	0.773	3 (0.9)	3 (1.0)	1.000	4 (1.6)	2 (0.8)	0.440
27. Is incontinent of feces	24 (4.2)	32 (5.6)	0.277	5 (1.5)	10 (2.2)	0.570	19 (7.6)	24 (9.2)	0.633
28. Throws food	3 (0.5)	2 (0.3)	1.000	3 (0.9)	0 (0.0)	0.249	0 (0.0)	2 (0.8)	0.499

*DBD, Dementia Behavioral Disturbance Scale; MMSE, Mini-Mental State Examination*.

**Table 3:** BPSD before and during COVID-19: results of multivariate analysis.

**Group/BPSD related symptoms**	**Unadjusted**		**Adjusted model**	
	**OR**	**95% CI**	**AOR**	**95% CI**
Mild group (MMSE 21–30)				
Wakes up at night for no obvious reason	1.89	1.09–3.28	1.82	1.02–3.23
Physical attacks	3.95	1.09–14.28	4.25	1.12–16.07
Moderate/severe group (MMSE 11–20)				
Wakes up at night for no obvious reason	2.16	1.39–3.34	1.96	1.19–3.23
Sleeps excessively during the day	1.47	1.04–2.09	1.45	0.93–1.05
Paces up and down	1.74	1.08–2.80	1.49	0.86–2.58
Wanders in the house at night	2.09	1.08–4.06	2.22	1.03–4.81
Gets lost outside	1.96	1.05–3.69	1.80	0.88–3.66

*In Adjusted model, the outcome was a BPSD-related variable; MMSE, long-term care, GDS, Need for financial support, polypharmacy, living status, IADL, and Education were included as covariates in a binomial logistic regression analysis*.

*AOR, adjusted odds ratio; BPSD, behavioral and psychological symptoms of dementia; CI, confidence interval; FRI, Fall Risk Index; GDS, Geriatric Depression Scale; IADL, instrumental activities of daily living; MMSE, Mini-Mental State Examination*.

In the subanalysis of the prevalence of BPSD in the first and second halves of the COVID-19 period, four items were significantly less prevalent in the mild group, specifically “Asks the same question over and over again,” “Loses, misplaces, or hides things,” “Sleeps excessively during the day,” and “Hoards things for no obvious reason” and two in the moderate/severe group, specifically “Paces up and down” and “Repeats the same action over and over again” (Supplementary Table). However, there was no difference in the items that were significant in the analysis of “before” and “during” using the chi-squared test.

## Discussion

The prevalence of behavioral and psychological symptoms showed a different trend after the onset of the COVID-19 pandemic in patients with cognitive decline. Differences in behaviors related to the severity of dementia were also observed. Prevalent BPSD, such as memory impairment and apathy, showed a similar proportion before and during the pandemic, whereas sleep disturbance and aggressiveness were observed to be more prevalent during COVID-19.

Among the BPSD, the prevalence of “Waking up at night for no obvious reason” was higher during the pandemic, regardless of the degree of dementia. Prior research suggests that with increasing restrictions on behavior, such as the declaration of a state of emergency, it is possible that the frequency of going out decreases (35), social interaction is reduced (11), necessary care services are not available (6), daytime napping increases and the amount of physical activity decreases, resulting in the disturbance of circadian rhythm (36). An analysis of this study's sample characteristics shows that the percentage of individuals who were physically active tended to be lower in the mild group, while the percentage of individuals who reported worsening sleep quality and increased napping was higher in the moderate/severe group. In terms of long-term care services, the proportion of individuals using day care tended to be lower during COVID-19, and the proportion of individuals using alternative care services (day care and home visits) was not relatively higher. Based on the results of previous studies (13, 37), we believe that these factors are involved in the occurrence or worsening of BPSD; however, an analysis based on social factors is desirable.

Cagnin et al. reported that sleep disorders were the most frequent new-onset BPSD during COVID-19, at 21.3%, and the frequency of sleep disorders increased as the dementia severity increased (13). Our results show a similar trend, with 12.2% in the Mild group vs. 28.9% in the moderate/severe group. They also indicate that the frequency is higher in emergencies than in normal times. Furthermore, a subanalysis of early and late periods during the pandemic showed that the prevalence of sleep disorders was 13.0% in the early period and 11.0% in the late period in the mild group, and 31.7 and 24.5% in the moderate/severe group, with neither differences being significant. Thus, the increased prevalence of sleep disorders reported after COVID-19 persists for a long time. Sleep disturbance is an important early sign of mental health problems in the elderly who have experienced major changes in their living environment or psychological trauma due to disasters (38–41). Sleep disturbances not only have a negative impact on patients with AD but also contribute to the physical and mental burden of the primary caregiver (42). Therefore, there is a need for a system to monitor sleep status at an early stage and link it to evidence-based programs to improve sleep, such as pharmacotherapy and cognitive behavioral therapy (43).

Other than sleep symptoms, only the symptom of “Physical attacks” appeared more prevalent after COVID-19 after adjusting for related factors. In the subanalysis of the Mild group, no difference was observed, suggesting that the symptoms persisted overtime during the pandemic. Relatedly, and interestingly, symptoms attributed to the relationship between caregivers and dementia patients, such as “Makes unwarranted accusation” and “Is verbally abusive, curses,” showed higher prevalence later in the subanalysis, suggesting the need to pay attention to these symptoms when emergencies are prolonged. For people with dementia, the impact of changes in their living environment is highly stressful, and verbal abuse and violence can occur in the early stages of cognitive decline, leading to a significant increase in caregiver burden (44, 45). Previous studies have reported that aggression associated with dementia often arises from rejection of care, when the person does not understand the need for care, or misinterprets the caregiver's intention (46). If the caregiver insists on providing care, the person with dementia defends himself or herself from this unwanted attention and may become combative. In the context of behavioral restrictions associated with COVID-19, it is possible that aggression increases as those with dementia and family caregivers spend more time at home and are less likely to receive care services, such as in day cares. Deterioration in the psychological state of both patients and caregivers may lead to a vicious cycle, with deterioration in the health and quality of life of both. To overcome this issue, online programs for social participation and psychological support have been developed, and online video platforms for non-pharmacological therapies (e.g., life review program), which are considered effective for patients with BPSD, are being used (47), possibly reducing patient distress. Dementia patients had difficulty receiving necessary care services (especially residential services) after COVID-19 emerged, and the use of other services (such as day-care services) as alternatives had not increased, suggesting that BPSD and care burden are currently increasing.

To date, only a few studies have been conducted on people with special needs, especially dementia (48). A study of 40 cognitively impaired patients assessed BPSD in the early stages of the COVID-19 pandemic using the NPI questionnaire: ~30% of the patients reported the worsening of BPSD, and apathy was prominent (37). Using a cross-sectional design after COVID-19, an 87 multicenter Italian study of family caregivers of dementia patients reported worsening apathy in 34.5% and new-onset apathy in 17.1% (13). Apathy is one of the most frequent BPSD symptoms from the early to late stages of AD (49), and apathy during MCI is considered a predictor of the transition to dementia (50). In the present study, there was no difference in the prevalence of apathy before and after COVID-19 in both the mild and moderate/severe groups. Although these results are seemingly contradictory, the study design differs in that the Italian study focused on changes at a single point after COVID-19, while this study focused on prevalence before and after the pandemic. Moreover, in the Italian study, caregivers were only asked about the presence or absence of each symptom of BPSD, and no quantitative assessment using a standardized scale was conducted. In addition, although the subjects of each study were patients of the memory clinic, the Italian study included caregivers of all patients who visited the clinic, whereas the present study included patients and their families who had made their first visit to the outpatient memory clinic, which may better reflect the current problems of the patients and their families. Although we cannot directly compare these studies on apathy in patients with dementia after COVID-19, the fact that this study reported a prevalence of ~40% in the mild group and 60% in the moderate/severe group suggests that patient condition is deteriorating and that apathy should be carefully considered in patients with dementia.

Another important BPSD is depressive symptoms. Tsugawa et al. evaluated 126 AD patients during the pandemic using the same GDS scale as in this study, and found that patients with severe AD were less aware of the spread of infection than patients with mild AD, and therefore also had lower depressive symptoms than patients with mild AD (12). However, we evaluated a larger number of patients over a longer period of time and found higher GDS scores in patients with moderate to severe AD, different from Tsugawa et al. Previous studies among the elderly in Japan have shown a negative association between MMSE and GDS scores, with a tendency toward depression as cognitive function declined (51), which is consistent with the results of this study. Although the previous study by Tsugawa et al. provides an important dataset from the early stages of COVID-19 (May–June 2020), we believe that the small sample size, differences based on dementia subtype, and gender distribution could explain the different findings compared to our study. Our data indicates that depressive symptoms and other psychological stresses should be addressed even in patients with moderate to severe AD.

## Strengths and Limitations

We examined the behavioral and psychological symptoms of patients with cognitive decline before and during the COVID-19 pandemic, using propensity score matching and adjusted models for possible confounders. As the results did not support cognitive decline similar to that reported in a previous study (11), and since we were unable to analyze the same subjects before and after the pandemic, we cannot refer to clear changes between the study periods; however, the results showed different BPSD symptoms expressed in the target group with the same degree of cognitive function. There are some limitations to this study. One is that the design of the study is not longitudinal, since we did not follow the same subjects from before COVID-19, it is not possible to track individual changes. In addition, because the study was conducted at a single institution and most of the subjects were accompanied by their family members, we cannot completely exclude information bias. Third, although a previous study pointed out that BPSD varied depending on the type of dementia (13), the present study has the limitation of including all dementia types within one single dementia group. In the future, it will be necessary to analyze each type of dementia with a larger number of cases, conduct follow-up longitudinal studies, as well as comparative studies in multiple facilities with different environments, such as urban and rural areas.

This study showed that the profile of behavioral and psychological symptoms in patients with cognitive decline differed in the individuals that were evaluated during the pandemic than in those evaluated before the pandemic, and some symptoms were associated with the severity of dementia. BPSD prevalence, such as memory impairment and apathy, showed similar profiles before and during the pandemic, whereas sleep disturbance and aggressiveness were more prevalent during the pandemic. The latter symptoms should be screened for during periods of disturbance and require evidence-based interventions.

## Data Availability Statement

The raw data supporting the conclusions of this article will be made available by the authors, without undue reservation.

## Ethics Statement

The studies involving human participants were reviewed and approved by Ethics Committee of the NCGG. The patients/participants provided their written informed consent to participate in this study.

## Author Contributions

YKu and TSa designed the study and planned recruitment. YKu performed statistical analyses and wrote first draft. TSu, NM, KU, YKi, CS, and TSa contributed to the interpretation and discussion of results and reviewed the manuscript. All authors contributed to the article and approved the submitted version.

## Funding

This study was financially supported by grants from the Research Funding of Longevity Sciences (21-28, 22-23) from the NCGG, and Japan Society for the Promotion of Science (JSPS) KAKENHI Grant Number 18K11779 (Principal investigator; YKu).

## Conflict of Interest

The authors declare that the research was conducted in the absence of any commercial or financial relationships that could be construed as a potential conflict of interest.

## Publisher's Note

All claims expressed in this article are solely those of the authors and do not necessarily represent those of their affiliated organizations, or those of the publisher, the editors and the reviewers. Any product that may be evaluated in this article, or claim that may be made by its manufacturer, is not guaranteed or endorsed by the publisher.
