# Supplementary material for: Prevalence of Behavioral and Psychological Symptoms in Patients With Cognitive Decline Before and During the COVID-19 Pandemic
Source: Front Psychiatry. 2022 Mar 7;13:839683. doi: 10.3389/fpsyt.2022.839683 (PMC8934776; doi:10.3389/fpsyt.2022.839683)
Supplement: Supplementary file 1 [file Data_Sheet_1.docx]

Supplemental Table: Proportion of patients with behavioral disorders in each item of DBD before and during COVID-19, by severity of cognitive impairment.

|  | Mild Group  (MMSE 21-30)  *n* (%) | | *P*-value | Moderate-Severe Group  (MMSE 11-20)  *n* (%) | | *P*-value |
| --- | --- | --- | --- | --- | --- | --- |
|  | First half | Second half |  | First half | Second half |  |
| 01. Asks the same question over and over again  02. Loses, misplaces, or hides things  03. Shows lack of interest in daily activities  04. Wakes up at night for no obvious reason  05. Makes unwarranted accusations  06. Sleeps excessively during the day  07. Paces up and down  08. Repeats the same action over and over again  09. Is verbally abusive, curses  10. Dresses inappropriately  11. Cries or laughs inappropriately  12. Refuses to be helped with personal care  13. Hoards things for no obvious reason  14. Moves arms or legs in a restless or agitated way  15. Empties drawers or closets  16. Wanders in the house at night  17. Gets lost outside  18. Refuses to eat  19. Overeats  20. Is incontinent of urine  21. Wanders aimlessly outside or in the house during the day  22. Makes physical attacks  23. Screams for no reason  24. Makes inappropriate sexual advances  25. Exposes himself/herself indecently  26. Destroys property or clothing  27. Is incontinent of feces  28. Throws food | 152 (82.6)  126 (68.5)  73 (39.9)  24 (13.0)  23 (12.5)  75 (40.8)  8 (4.3)  13 (7.1)  23 (12.5)  17 (9.2)  8 (4.4)  30 (16.3)  46 (25.1)  5 (2.7)  7 (3.8)  5 (2.7)  7 (3.8)  5 (2.7)  20 (10.9)  15 (8.2)  4 (2.2)  6 (3.3)  5 (2.7)  0 (0)  0 (0)  0 (0)  4 (2.2)  0 (0) | 93 (72.1)  73 (56.6)  52 (40.6)  14 (11.0)  27 (20.9)  35 (27.1)  9 (7.0)  13 (10.1)  26 (20.2)  12 (9.3)  9 (7.0)  21 (16.3)  19 (14.8)  5 (3.9)  5 (3.9)  4 (3.1)  5 (3.9)  4 (3.1)  14 (10.9)  11 (8.6)  3 (2.3)  5 (3.9)  4 (3.1)  0 (0)  0 (0)  3 (2.3)  3 (2.3)  0 (0) | **0.036**  **0.033**  0.907  0.725  0.059  **0.016**  0.323  0.407  0.082  1.000  0.325  1.000  **0.033**  0.749  1.000  1.000  1.000  1.000  1.000  1.000  1.000  0.764  1.000  –  –  0.068  1.000  – | 150 (91.5)  135 (82.3)  107 (65.6)  51 (31.7)  46 (28.0)  87 (53.0)  40 (24.5)  41 (25.2)  32 (19.5)  50 (30.9)  20 (12.3)  44 (26.8)  57 (34.8)  16 (9.8)  25 (15.3)  22 (13.5)  22 (13.5)  11 (6.7)  34 (21.0)  34 (20.7)  23 (14.1)  8 (4.9)  6 (3.7)  1 (0.6)  1 (0.6)  2 (1.2)  17 (10.4)  1 (0.6) | 88 (90.7)  81 (84.4)  54 (56.8)  23 (24.5)  21 (21.9)  47 (49.0)  13 (13.5)  13 (13.5)  22 (22.9)  22 (22.9)  9 (9.4)  29 (30.2)  28 (29.2)  9 (9.4)  11 (11.5)  7 (7.4)  9 (9.4)  3 (3.1)  14 (14.6)  16 (16.7)  9 (9.4)  3 (3.1)  2 (2.1)  1 (1.0)  0 (0)  0 (0)  7 (7.3)  1 (1.0) | 0.825  0.734  0.183  0.254  0.306  0.607  **0.038**  **0.027**  0.529  0.197  0.545  0.570  0.412  1.000  0.459  0.156  0.428  0.265  0.247  0.515  0.330  0.751  0.714  1.000  1.000  0.532  0.508  1.000 |

Abbreviations: DBD = Dementia Behavioral Disturbance Scale, MMSE = Mini-Mental State Examination.

Supplemental Figure: Flow-chart of selection of participants to be analyzed.
